# The association between violence against women and chronic pain: a systematic review and meta-analysis

**DOI:** 10.1186/s12905-024-03097-w

**Published:** 2024-06-04

**Authors:** Allison Uvelli, Carola Ribaudo, Giacomo Gualtieri, Anna Coluccia, Fabio Ferretti

**Affiliations:** 1 Department of Medical Science, Surgery and Neurosciences, University of Siena, Viale Bracci, Siena, 53100 Italy; 2 AOUC Azienda Ospedaliero-Universitaria Careggi, Largo Brambilla, Florence, 50134 Italy; 3 AOUS Azienda Ospedaliero-Universitaria Senese, Viale Bracci, Siena, 53100 Italy

**Keywords:** Violence against women, Chronic pain, Systematic review

## Abstract

Violence against women is a phenomenon that involves at least 35% of women worldwide. Violence can be sexual, physical, and/or psychological, perpetrated by the partner, another family member, or a stranger. Violence is a public health problem because its consequences include higher morbidity, higher mortality, and short and long-term physical and psychological health diseases. Most studies prove an association between any type of violence and some chronic pain diagnoses but no one has done a complete collection of this evidence. This systematic review and meta-analysis aimed to evaluate whether this association is statistically significant, including the largest number of studies. Through the inclusion of 37 articles, the association has been demonstrated. Compared with no history of violence, women who did experience violence showed 2 times greater odds of developing chronic pain. The impact of violence was significant also on fibromyalgia separately, but not on pelvic pain.

**PROSPERO registration**

PROSPERO CRD42023425477.

## Background

### The phenomenon of gender violence

Violence against women is a violation of their human rights and a public health issue [1]. This violence can take many forms, such as interpersonal violence, domestic abuse, or intimate partner violence, including sexual, physical, and psychological violence. The ultimate goal is to exert control over women's lives, which can destroy their independence, self-determination, personal growth, and psychological well-being.

The United Nations [2] defines violence against women as “any act of gender-based violence that results in or is likely to result in, physical, sexual or mental harm or suffering to women, including threats to such acts, coercion or arbitrary deprivation of liberty, whether occurring in public or private life”. In recent years, policies aimed at addressing and preventing violence have been on the rise. However, despite these efforts, the incidence of violence remains high. According to a recent observational study, approximately 51.7% of women in the European Union have experienced violence at some point in their lives [3]. Additionally, at least 35% of women worldwide have reported experiencing violence [4].

Intimate partner violence (IPV) is the most prevalent type of violence against women, and it is more difficult to detect and study than violence perpetrated by strangers [5]. IPV is defined by the Centers for Disease Control and Prevention (CDC) as violence perpetrated by a current or former intimate partner, including physical violence, sexual violence, stalking, and psychological aggression [6]. In the last decade, there has been a significant amount of literature on this topic, but there are still many aspects that require further exploration.

### Consequences of abuse

Abuse survivors suffer various consequences that range from higher morbidity and mortality and short and long-term physical and psychological health problems [7]. Victimized women have higher rates of depression, post-traumatic stress disorder (PTSD), anxiety, alcoholism, and suicidal tendencies [8–10].

Apart from psychological consequences, violence can cause chronic health problems and pain [11–14]. The prevalence of chronic pain is 8.78% in Europe [15], 6% in Canada [16], and 8% in the United States of America [17]. Most studies focus on pelvic/vaginal pain [18], fibromyalgia [19], irritable bowel syndrome/bowel symptoms [20], abdominal pain [21], temporomandibular pain [22], breast pain [23], migraine/headache [24], back pain [25], and neck pain [26], which tend to become chronic as well as the pain derived from them.

Previously, researchers studied health conditions and violence types separately, such as the association between sexual trauma and pelvic pain [27, 28], or psychological trauma and fibromyalgia [29, 30]. It is crucial to approach chronic pain as a broad concept because irrespective of the affected body area, individuals can seek assistance from pain therapy departments or clinics. Therefore, it would be beneficial to conduct a general screening.

Several studies and systematic reviews have examined the link between adverse childhood experiences (ACE) and chronic pain [31, 32]. Additionally, some studies have investigated the relationship between post-traumatic stress disorder (PTSD) and physical health consequences [33, 34]. There are also longitudinal studies that track the health of abused women over time [35–37]. Most of these studies show a positive association between any kind of violence and all chronic conditions, with a significant difference between the group of abused women and the group of not-abused women [38–44]. Despite this, the exact cause of these conditions is still not clear.

Given the complexity of the situation at hand, the bio-psycho-social model [45] could provide us with a better understanding of the underlying causes. This model asserts that diseases arise from a combination of biological, psychological, and social factors that vary from person to person. Accordingly, if we adopt this approach, we could infer that chronic pain in victims of violence is not exclusively caused by a single factor, be it psychological, sociological, or biological, but rather by a combination of all three.

### What is the current stage of gender research?

Studies on gender violence have been conducted for many years, and they continue to be relevant in recent times. These studies have created a large database that experts and researchers can use to orient their study or clinical practices. It is essential to categorize available data into various evidence collections to have more precise estimates. In particular, data on the health of abused women can guide health policies and guidelines. It can also help healthcare professionals in their clinical work and better direct women toward proper treatment. However, there are no reviews that consider chronic pain as a general concept in this regard. This is the first meta-analysis that investigates the association between violence against women in adulthood and chronic pain.

## Objectives

This systematic review and meta-analysis aim to contribute to the clarification of the literature on the association between violence against women and chronic pain, considering all types of abuse verified only in adulthood (abuse suffered after 18 years old), and the major chronic pain diagnoses found in the literature.

The final objective is to increase the knowledge about this condition because no one has done this complete collection before considering it as a wide-ranging phenomenon.

## Materials and methods

This search protocol was based on the Preferred Reporting Items for Systematic Reviews and Meta-Analysis (PRISMA) guidelines [46], according to the PECOS (Population, Exposure, Comparison, Outcome, Study Design) guidelines.

### Search strategy

The research was conducted on the online electronic databases of PubMed, Scopus, and Web of Science from October 2022 to May 2023, and carried out a manual review of references. The databases are selected to contain the most high-quality empirical studies. The protocol has been registered at the International Prospective Register of Systematic Reviews (PROSPERO; registration number CRD42023425477).

The search strategy relating to the association between violence against women and chronic pain was: (“intimate partner violence” OR “interpersonal violence” OR “partner abuse” OR “domestic violence”) AND ((pain)). The keywords have been chosen after a preliminary search of the literature thanks to which it was possible to identify the most used and relevant terms. The term “violence against women” is not included due to its generalizable meaning, risking losing the focus of the research because it resulted in too many irrelevant articles.

There were no period restrictions on the search to increase the yield of studies, though the language was restricted to studies published in English or Italian.

Authors were also contacted via email where there was insufficient data, and references from included studies were manually scanned for further sources as per published recommendations [47–49].

### Criteria for selection of studies

Studies were included if they met the following criteria: human females of at least 18 years old with (“cases”) and without (“controls”) a history of adulthood abuse identified through published observational study designs (cohort, case–control, and cross-sectional studies). For the definition of abuse, we did not limit inclusion criteria based on specific types of violence. Definitions of chronic pain varied between studies and it also adopted an inclusive approach. In general, the pain or discomfort that persists in the affected area for at least 3 out of the past 6 months. Pain signs and symptoms considered were pelvic pain, fibromyalgia, bowel pain, abdominal pain, temporomandibular pain, breast pain, migraine/headache, back pain, and neck pain. Exclusion criteria included male subjects, minor subjects, childhood abuse (abuse suffered before 18 years old), studies without a control group, and studies not published in English or Italian languages. Only the relevant sample was extrapolated from articles with mixed samples.

Lastly, systematic reviews, meta-analyses, commentaries, dissertations, thesis, editorials, and conference deeds were excluded but their references were examined to find other studies not retrieved between the search strategy.

### Study selection and data extraction

Studies were selected in a three-stage process. All citations identified from initial searching (articles extracted in October 2022) were imported into Zotero Software, where duplicate citations were removed, after which two reviewers (AU & CR) independently scrutinized all article titles remaining from the original search. After this, the same two reviewers independently analyzed all article abstracts remaining from the second removal. In case of disagreement, the references were discussed until an agreement was reached, and an independent third reviewer (FF) was consulted. In case of unclear abstract, the reference was included in the next stage (full-text screening) to confirm the information given in the full text. For studies assessed for eligibility full manuscripts were obtained, and two reviewers (AU & CR) carried out an independent full-text review of all English/Italian language articles. Disagreements regarding inclusion or exclusion criteria were resolved by consensus, or through consultation of an independent third reviewer (FF). Two reviewers (AU & CR) carried out independent data extraction, where extractable data was missing, authors were contacted by email. It used outcome data and exposure to abuse to construct a table for the appropriate analyses.

### Assessment of study quality

Quality assessment was conducted through existing checklists [50]. Quality was defined as the confidence that bias in the estimation of the effect of abuse on pain symptom outcomes was minimized through appropriate study design methods and analysis. Two independent authors (AU & CR) assessed the quality of the retrieved articles to identify any potential source of bias using predetermined and validated criteria from The Joanna Briggs Institute appraisal checklists for cross-sectional, case–control, and cohort studies [50]. Appraisal criteria are made of comparability and appropriateness of cases and controls, description of subjects and setting, reliable and valid measurement of exposure, appropriateness of inclusion criteria, identification of confounding factors and whether strategies were implemented to deal with these factors, valid and reliable assessment of outcomes, exposure time, appropriateness of follow-up and whether strategies were implemented to deal with incomplete follow-up, and appropriateness of statistical analyses used. To ensure the quality of a study, certain criteria must be met. For cross-sectional studies, at least 5 out of 8 criteria should be met, while cohort studies should meet at least 6 out of 11 criteria. Case–control studies should meet at least 6 out of 10 criteria. Any study that fails to meet these standards will be considered low-quality and excluded from the results. In this particular study, 10 cross-sectional studies satisfied 6 out of 8 criteria, 14 satisfied 5 out of 8 criteria, and 1 satisfied all 8 criteria. Of the cohort studies, 7 out of 11 criteria were met. For the case–control studies, 2 satisfied 6 out of 10 criteria, 2 satisfied 8 out of 10 criteria, 2 satisfied 7 out of 10 criteria, and 1 satisfied all 10 criteria. All articles were found to meet the high-quality criteria, therefore, no articles were excluded based on the quality standard.

### Statistical analysis

Statistical analyses were performed using Comprehensive Meta-Analysis Software (CMA) v4. Since the prevalence of chronic pain could be affected by the characteristics of the populations included, and considering the heterogeneity of the symptoms defining this health problem, random-effect models were used in this study [51]. The effect size was assessed in the odds ratio of having chronic pain and being a victim of violence. The results of three meta-analyses were evaluated: chronic pain in general, pelvic pain, and fibromyalgia. The two sub-categories of pelvic pain and fibromyalgia were also evaluated individually because they had a sufficient number of studies to be able to do it. The effect sizes were estimated by the odds ratios’ 95% confidence interval. Forest plots were generated, and heterogeneity analysis of the effect sizes was performed by calculating Higgins’s *I*^2^ statistic [52] and Cochrane’s *Q* index [53]. Cochrane’s *Q* *p* value < 0.1 and an *I*^2^ > 40% were considered markers of heterogeneity.

Publication bias was explored through the inspection of the funnel plot and the Egger test [54]. The funnel plot appears asymmetrical if publication bias is detected, while a non-statistically significant result of the t-value of Egger’s regression intercept allows us to discard publication bias. The level of significance was set at *p* < 0.05.

## Results

### Literature identification, study characteristics, and quality

**Fig. 1:**
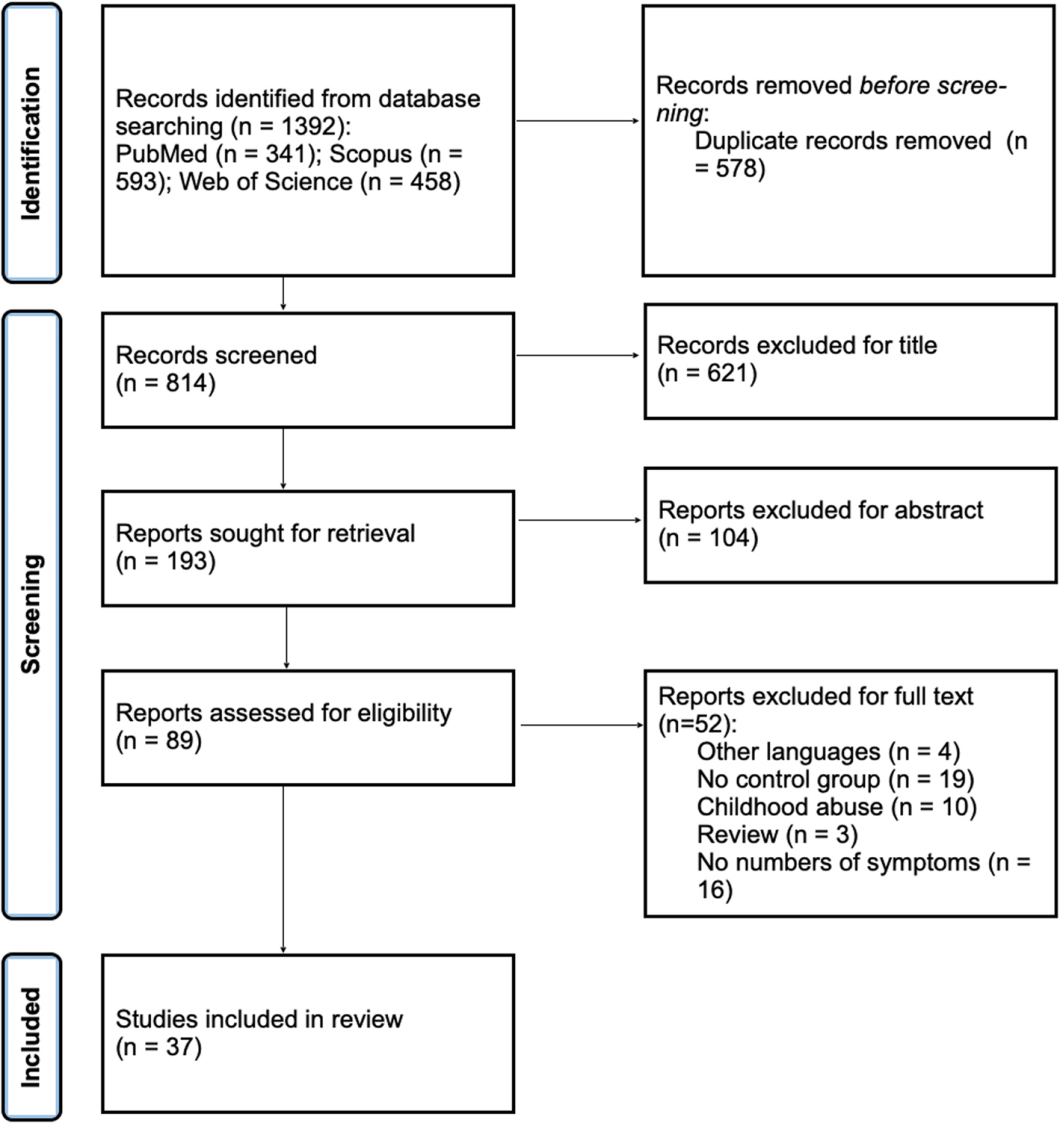
Prisma flowchart [46]

The search protocol identified 1392 publications from online databases. 578 were removed as they were duplicate publications. The remaining 814 studies were screened against title and abstract criteria, after which a further 725 were excluded. Of the 89 studies selected for full-text review, 52 were excluded, 4 were written in unknown languages, 19 were due to lack of a control group, 10 focused on childhood abuse, 3 were review, and 16 were due to lack of some important data (the specific number of women with chronic pain in both groups, victimized and not victimized). After the quality assessment was carried out 37 studies were included [12, 23, 25, 55–88]. See the flow diagram in Fig. 1.

The years of the study range from 1994 to 2021, 26 studies are cross-sectional, 7 are case–control, and 4 are cohort studies. Studies from the USA are 56.7%, 10.8% are from the UK, 5.4% are from Spain and Australia respectively, and 21.5% are from other countries. The sample size ranges from 50 to 92,735, its age ranges from 18 to 65, and all the adult abuse types are represented. Studies evaluated general chronic pain are 56.7%, 18.9% evaluated fibromyalgia, and 24.3% evaluated pelvic pain. Studies evaluated intra-family violence (perpetrated by partners or other family members) are 78.3%, and 21.6% evaluated extra-family violence. Only physical, sexual, and psychological violence were examined in the included studies. Table 1 summarizes the characteristics of the included studies.

**Table 1:** Characteristics of the included studies

Authors	Controls	Cases	Study design	Type of violence	Meta-analysis inclusion group
Talley et al. (1994) Australia [55]	111 30–49 y/o 35 with pain	89 30–49 y/o 45 with pain	Cross-sectional study	Spousal violence	Gastrointestinal chronic pain
Boisset-Pioro et al. (1995) Canada [56]	132 51.2 y/o 39 with pain and fibromyalgia	112 49.3 y/o 44 with pain and fibromyalgia	Case–control study	Sexual and physical abuse	Fibromyalgia and any chronic pain
Taylor et al. (1995) USA [57]	47 49.5 y/o 11 with pain 20 with fibromyalgia	35 44.58 y/o 17 with pain 20 with fibromyalgia	Case–control study	Sexual and physical abuse	Fibromyalgia and any chronic pain
Letourneau et al. (1999) USA [58]	151 33 y/o 37 with pain	40 33 y/o 17 with pain	Cross-sectional study	Sexual, physical and emotional abuse	Any chronic pain
Coker et al. (2000) USA [59]	532 18–65 y/o 118 with pain 50 with pelvic pain	620 18–65 y/o 235 with pain 107 with pelvic pain	Cross-sectional study	Intimate Partner Violence	Pelvic pain and any chronic pain
Dienemann et al. (2000) USA [60]	30 25–45 y/o 8 with pain	47 25–45 y/o 21 with pain	Cross-sectional study	Domestic violence	Chronic headache
Lown & Vega (2001) USA [61]	1057 32 y/o 6 with pain	126 32 y/o 13 with pain	Cross-sectional study	Intimate Partner Violence	Back chronic pain
Weinbaum et al. (2001) USA [62]	2776 18–65 y/o 571 with pain	207 18–65 y/o 73 with pain	Cross-sectional study	Intimate Partner Violence	Any chronic pain
Campbell et al. (2002) USA [63]	1000 21–56 y/o 252 with pain 86 with pelvic pain	980 21–56 y/o 389 with pain 169 with pelvic pain	Case–control study	Intimate Partner Violence	Pelvic pain and Any chronic pain
Kramer et al. (2004) USA [64]	718 18–65 y/o 167 with pain	550 18–65 y/o 291 with pain	Cross-sectional study	Intimate Partner Violence	Any chronic pain
John et al. (2004) UK [65]	649 20–60 y/o 86 with pain	171 20–60 y/o 36 with pain	Cross-sectional study	Physical domestic violence	Lower abdominal chronic pain
Castro et al. (2005) USA—Guatemala [66]	257 44.5 y/o 67 with pain 30 with fibromyalgia	117 44.5 y/o 62 with pain 28 with fibromyalgia	Case–control study	Physical, verbal and sexual abuse	Fibromyalgia and any chronic pain
Coker et al. (2005) USA [67]	530 18–65 y/o 21 with pain	624 18–65 y/o 60 with pain	Cross-sectional study	Intimate Partner Violence	Back chronic pain
Johnson et al. (2006) USA [23]	786 47 y/o 269 with pain	433 47 y/o 164 with pain	Cross-sectional study	Domestic violence	Breast chronic pain
Salam et al. (2006) Bangladesh—Japan [68]	132 25.4 y/o 48 with pain 31 with pelvic pain	364 25.4 y/o 189 with pain 128 with pelvic pain	Cross-sectional study	Spousal violence	Pelvic pain and any chronic pain
Pikarinen et al. (2007) Finland [12]	402 42.5 y/o 79 with pain	289 42.5 y/o 92 with pain	Cross-sectional study	Physical and sexual abuse	Lower abdominal chronic pain
Plante & Kamm (2008) France—UK [69]	56 40 y/o 10 with pain and pelvic pain	110 34 y/o 68 with pain and pelvic pain	Cohort study	Sexual and physical assault, intra-family violence	Pelvic pain and any chronic pain
Ellsberg et al. (2008) Switzerland—USA—UK [70]	10,299 18–50 y/o 2165 with pain	9218 18–50 y/o 2856 with pain	Cross-sectional study	Intimate Partner Violence	Any chronic pain
Kendall-Tackett et al. (2008) USA [71]	53 47 y/o 21 with pain	57 47 y/o 30 with pain	Cross-sectional study	Domestic abuse	Chronic headache
Bonomi et al. (2009) USA [72]	1686 18–64 y/o 340 with pain 128 with pelvic pain	242 18–64 y/o 76 with pain 29 with pelvic pain	Cross-sectional study	Physical and sexual abuse	Pelvic pain and any chronic pain
Ruiz-Perez et al. (2009) Spain [73]	306 40.7 y/o 139 with pain and fibromyalgia	268 47.76 y/o 148 with pain and fibromyalgia	Case–control study	Physical, sexual and emotional abuse	Fibromyalgia and any chronic pain
Vung et al. (2009) Vietnam—Sweden [74]	802 18–60 y/o 81 with pain	81 18–60 y/o 24 with pain	Cross-sectional study	Intimate Partner Violence	Any chronic pain
Becker-Dreps et al. (2010) USA—Nicaragua [75]	776 37 y/o 104 with pain	186 37 y/o 47 with pain	Cross-sectional study	Intimate Partner Violence	Gastrointestinal chronic pain
Vives-Cases et al. (2011) Spain [25]	12,707 18–50 y/o 3069 with pain	278 18–50 y/o 109 with pain	Cross-sectional study	Intimate Partner Violence	Back chronic pain
Prosman et al. (2012) Netherland [76]	50 19–60 y/o 19 with pain 15 with pelvic pain	50 19–60 y/o 22 with pain 28 with pelvic pain	Case–control study	Intimate Partner Violence	Pelvic pain and any chronic pain
Eldoseri et al. (2014) UK [77]	111 18–65 y/o 35 with pain	89 18–65 y/o 45 with pain	Cross-sectional study	Spousal violence	Any chronic pain
Al-Modallal (2016) Jordan [78]	87 32.7 y/o 57 with pain 42 with fibromyalgia	151 32.7 y/o 120 with pain 99 with fibromyalgia	Cross-sectional study	Intimate Partner Violence	Fibromyalgia and any chronic pain
Halpern et al. (2016) USA [79]	30 18–64 y/o 18 with pain 0 with pelvic pain	34 18–64 y/o 26 with pain 3 with pelvic pain	Cross-sectional study	Intimate Partner Violence	Pelvic pain and orofacial chronic pain
Lacey & Mouzon (2016) USA [80]	836 18–65 y/o 129 with pain	113 18–65 y/o 30 with pain	Cross-sectional study	Intimate Partner Violence	Rhematic chronic pain
Ford et al. (2017) USA [81]	16 24.9 y/o 4 with pain	34 34.9 y/o 24 with pain	Cross-sectional study	Intimate Partner Violence	Any chronic pain
Iverson et al. (2017) USA [82]	47 39.8 y/o 12 with pain	80 36 y/o 36 with pain	Cross-sectional study	Intimate Partner Violence	Back chronic pain
Campbell et al. (2018) USA [83]	356 27 y/o 311 with pain	534 27 y/o 476 with pain	Case–control study	Intimate Partner Violence	Any chronic pain
Chandan et al. (2019) UK [84]	74,188 36.9 y/o 1507 with pain	18,547 36.9 y/o 343 with pain	Cohort study	Intimate Partner Violence	Temporomandibular joint chronic pain
Craner et al. (2020) USA [85]	48 46.87 y/o 1 with pain 6 with fibromyalgia 4 with pelvic pain	60 45 y/o 11 with pain 15 with fibromyalgia 0 with pelvic pain	Cross-sectional study	Intimate Partner Violence	Fibromyalgia, pelvic pain and any chronic pain
FitzPatrick et al. (2020) Australia [86]	1112 31 y/o 563 with pain 284 with pelvic pain	234 31 y/o 139 with pain 79 with pelvic pain	Cohort study	Intimate Partner Violence	Pelvic pain and any chronic pain
Chandan et al. (2021) UK [87]	74,188 36.9 y/o 53 with pain 239 with fibromyalgia	18,547 36.9 y/o 19 with pain 97 with fibromyalgia	Cohort study	Intimate Partner Violence	Fibromyalgia and any chronic pain
Trivedi et al. (2021) USA [88]	366 47.7 y/o 64 with pain	222 45.9 y/o 67 with pain	Cross-sectional study	Physical, sexual and emotional abuse	Chronic headache

### Meta-analysis results

**Fig. 2:**
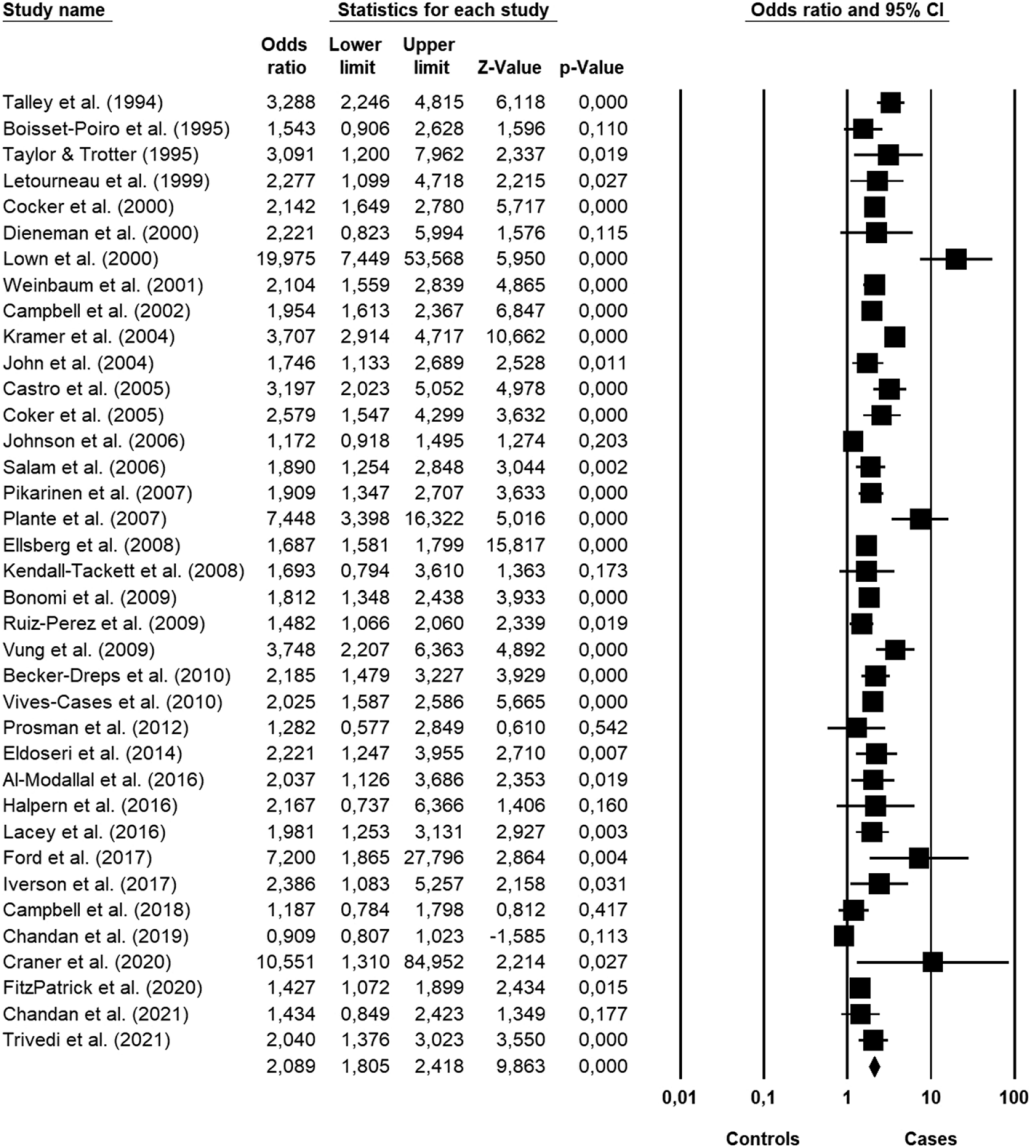
Forest plot showing the meta-analysis results about chronic pain in general

**Fig. 3:**
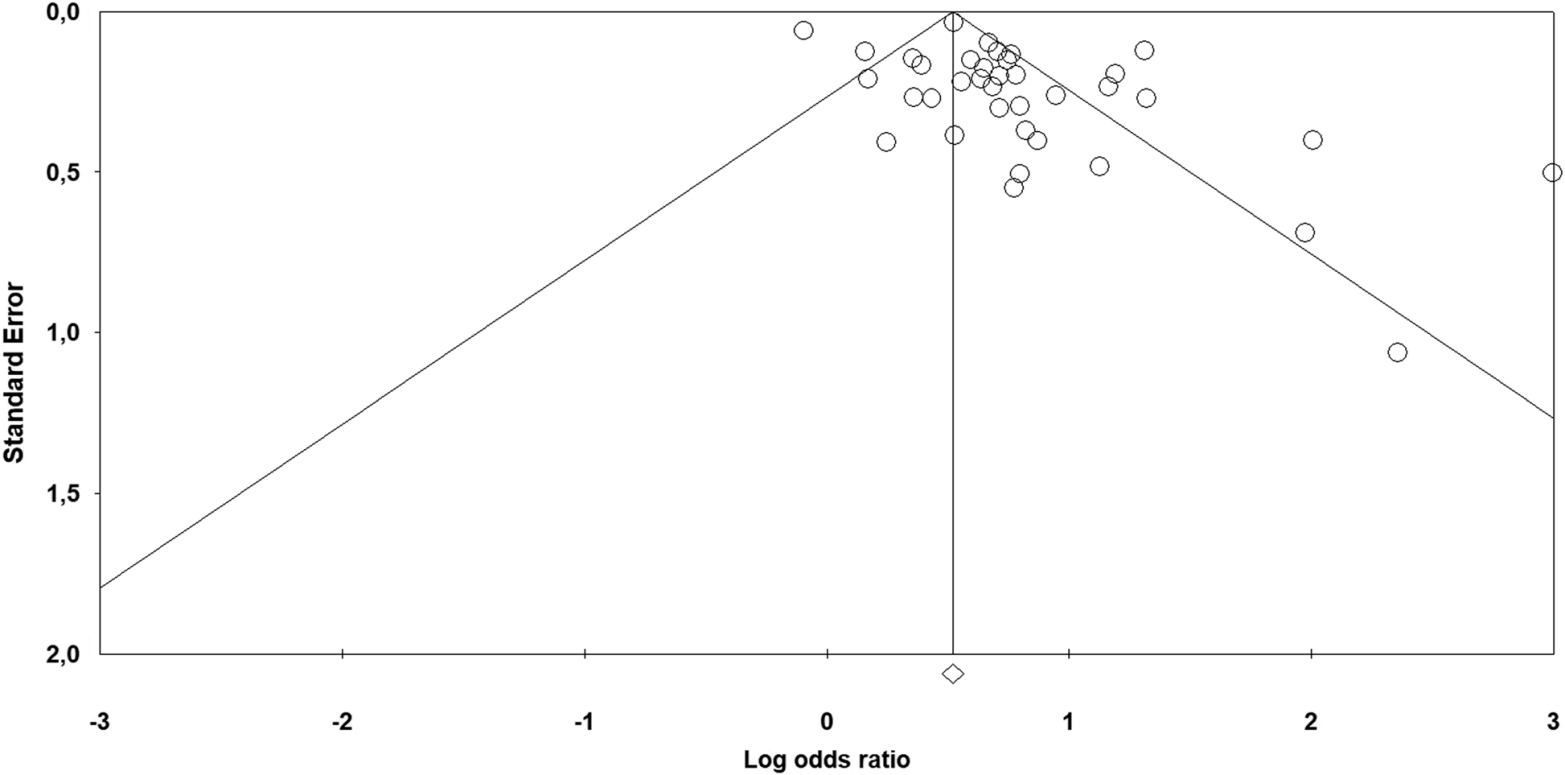
Funnel plot of chronic pain in general

Figure 2 shows the results of the random effects meta-analysis about the association between violence and chronic pain. Compared with no history of violence, women who did experience violence showed an increased odds of developing chronic pain (OR 2.08; 95% CI, 1.80–2.41; *p* < 0.001). Significant heterogeneity was found [*I*^2^ = 85.86, Q_(36)_ = 254.56, *p* < 0.001], and the existence of publication bias was proved by the Funnel plot (7 out of 37 studies were located on the right side out of the plot) (Fig. 3) and by the significance of the Egger test on regression intercept [β = 1.676, SE = 0.589, t_(35)_ = 2.844, *p* < 0.007].

**Fig. 4:**
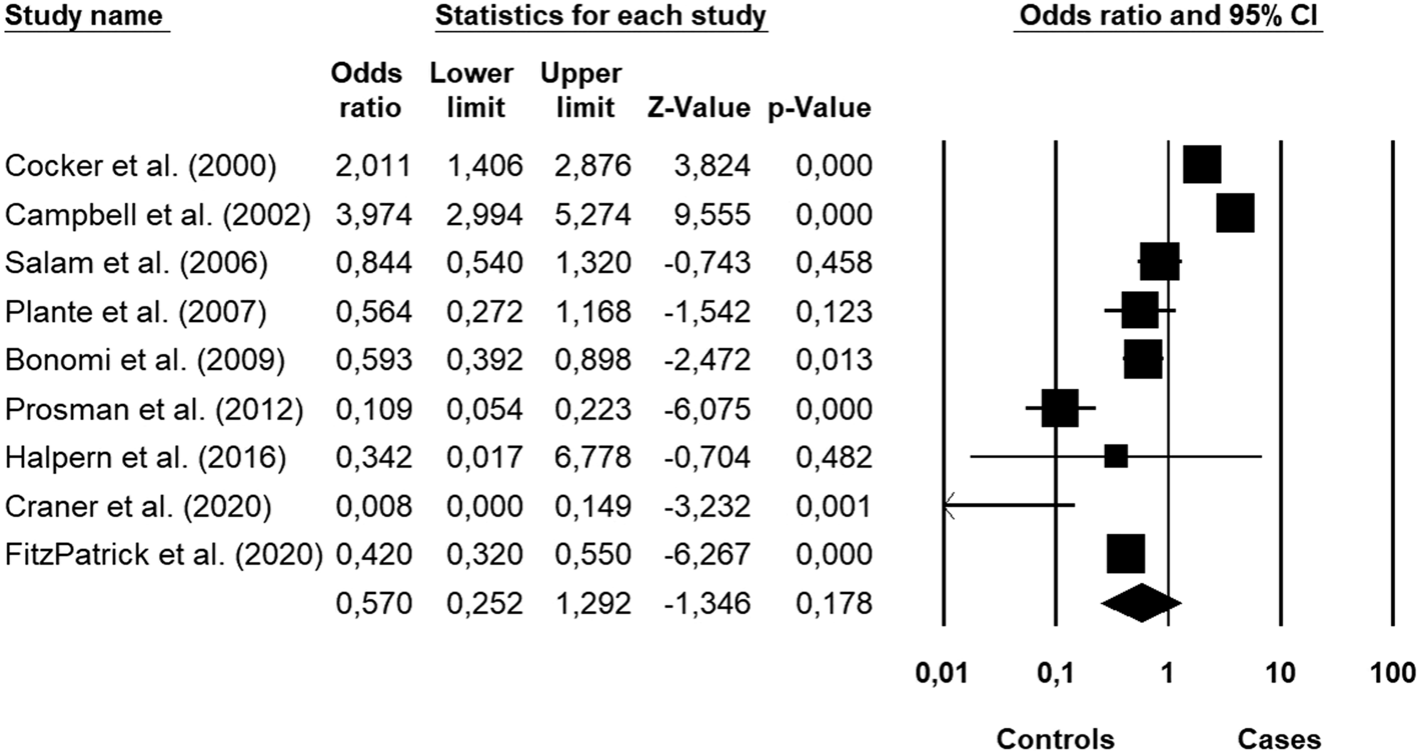
Forest plot of the meta-analysis results about pelvic pain

**Fig. 5:**
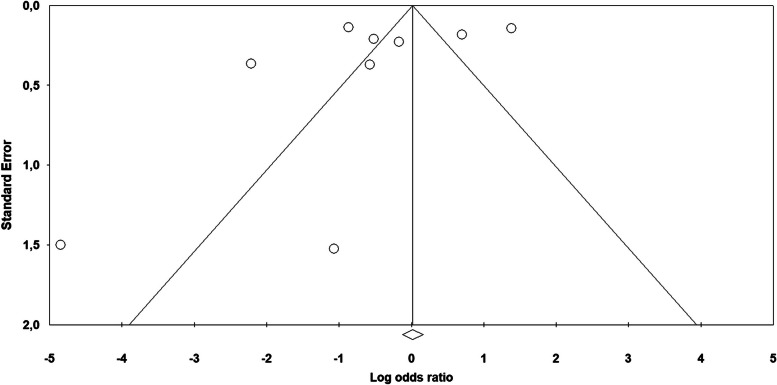
Pelvic pain Funnel plot

The subcategory of studies that investigated only pelvic pain showed a non-significant effect size. (OR 0.57; 95% CI, 0.25–1.29; *p* = 0.178). The forest plot with mean effect sizes is provided in Fig. 4. Significant heterogeneity was found [*I*^2^ = 96.040, Q_(8)_ = 2020.011, *p* < 0.001], but no publication bias was detected according to the Funnel plot and the Egger test of intercept (Fig. 5) [β = -3.657, SE = 3.284, t_(7)_ = 1.114, *p* < 0.302].

**Fig. 6:**
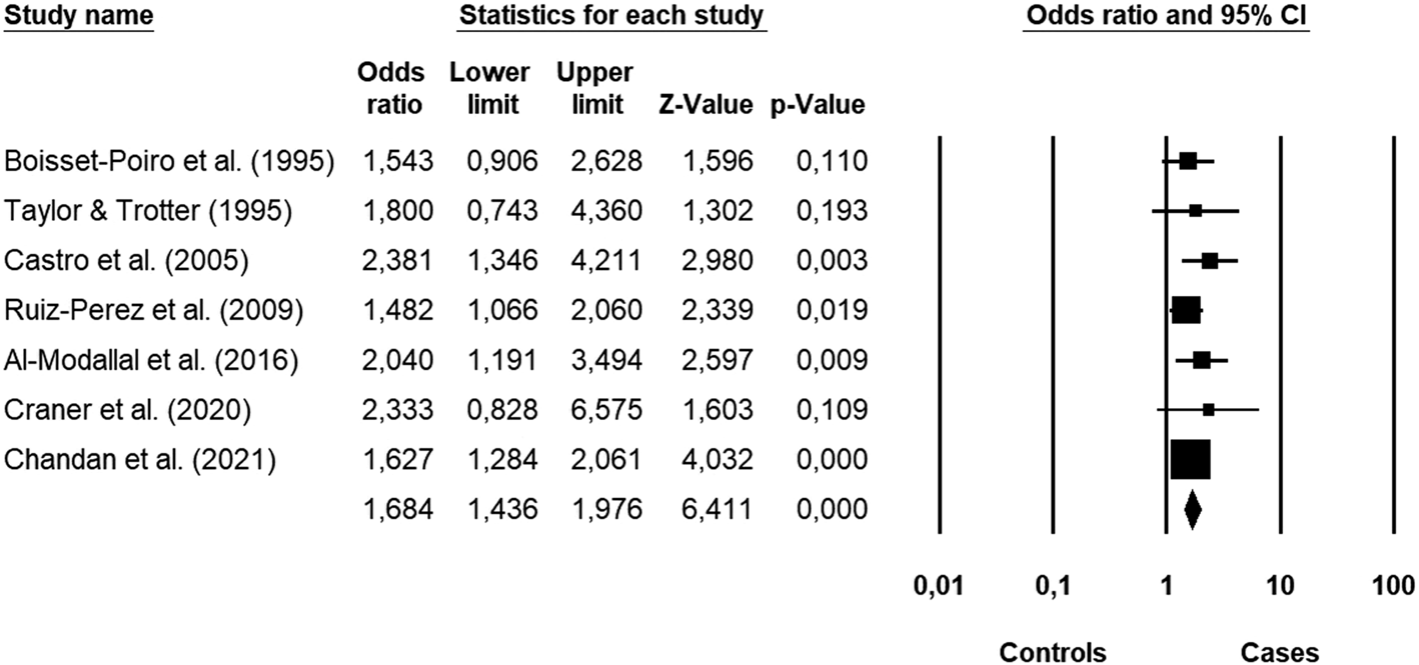
Forest plot of the meta-analysis results about fibromyalgia

**Fig. 7:**
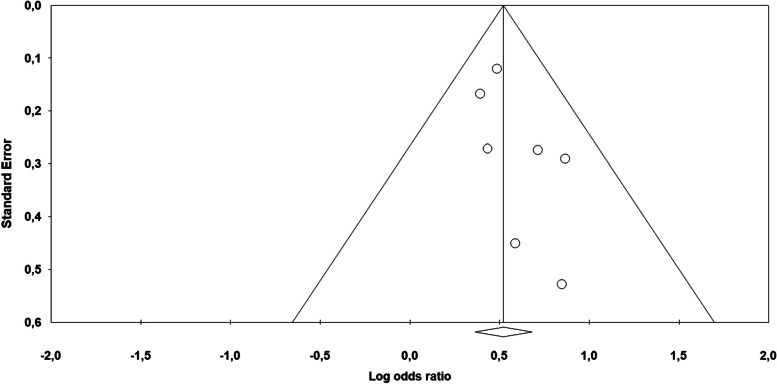
Fibromyalgia funnel plot

The impact of violence on fibromyalgia was significant (Fig. 6): the overall odds ratio effect size was 1.68 (95% CI: 1.44–1.98) with a *p* < 0.000. The forest plot highlighted a high level of homogeneity among the studies’ results [*I*^2^ = 0.000, Q_(86_ = 3.070, *p* < 0.800], together with the lack of publication bias: all the studies were located inside the Funnel (Fig. 7) and the Egger test of the intercept was not significant [β = 0.913, SE = 0.522, t_(5)_ = 1.748, *p* < 0.141].

## Discussion

### An healthcare point of view

In this review, we evaluated 37 studies on women both with and without a history of sexual, physical, and emotional abuse perpetrated by a partner, a family member, or a stranger. Results found strong and consistent associations between violence and the presence of chronic pain conditions. The results show that women who have experienced violence during adulthood have 2 times greater odds of developing chronic pain. Our findings are consistent with reports that women experiencing violence have adverse health outcomes [18–21], and with other systematic reviews that associate some pain symptoms/syndromes with some type of adult or childhood abuse [30–34]. Healthcare workers who treat women dealing with chronic pain should be aware that their patients may have experienced violence. Similarly, anti-violence workers should know that women who have suffered violence may develop chronic pain symptoms. Without collaboration and integration between these professionals, the treatment of such women may not be effective. Healthcare workers and anti-violence workers need to work together to provide holistic care and support to these women. Screening women with chronic pain may help identify hidden violence for health workers. Health policies, guidelines, and prevention practices also should consider this association to direct victims to a bio-psycho-social individualized cure. The path to ending violence must address both short and long-term health consequences, not just eliminate the problem. Unfortunately, the etiology of these conditions isn’t clear [89], despite longitudinal studies showing how pain persists even four years after separation from the abusive partner [90]. Furthermore, future research is needed to examine also associations between chronic pain and other forms of violence against women, such as financial abuse, stalking, cyber abuse, and coercion.

### The problem of an unclear etiology

According to the bio-psycho-social model [45], we may suppose many different factors that contribute to the onset of pain symptoms in violence victims, in fact, this condition is often associated with mental health problems, such as anxiety or depression [8–10], and social problems, such as lack of social support, but for each woman, the three types of factors have different importance. Mood disorders are often present in chronic pain patients with a bidirectional relationship, in fact, people affected by chronic pain were 2.0 to 2.5 more likely to experience an episode of depression than individuals without chronic pain [91, 92], and pain-free individuals with depressive disorder were 4 times more likely to develop chronic pain than not depressed individuals [93, 94]. The same relationship was obtained for all anxiety disorders [95–97], alcohol, drugs, and smoking use/abuse [98–100], and suicidal thoughts [101, 102]. Previous studies found a relationship between chronic pain and specific psychological aspects [103], and neuroimaging studies showed that the activated brain areas by nociceptive stimuli are the same ones involved in emotional and behavioral states [104]. Chronic pain is different from acute pain because it is not necessarily caused by injuries, though it still involves biological processes. Various sensory, autonomic, endocrine, and immune responses interact to contribute to the nociceptive stimuli that are perceived [105]. The nervous system is responsible for detecting potential threats, signaling danger to the body, and initiating a response to them. Similarly, the endocrine system helps in increasing the survival chances by causing an arousal response through the stress response. Lastly, the immune system detects microbial invasion and toxins and triggers complex inflammatory responses to fight them [106]. These processes could collectively weaken the body’s natural defense mechanism against pain. Persistent pain can result from locally inflamed processes that respond maladaptively to systemic changes at the nervous, endocrine, and immune levels. Chronic pain can persist even after the affected tissues have healed, due to abnormal sensory processes [105]. Trauma, such as violence and abuse, can trigger the autonomic nervous system and the immune system, leading to an inflammatory response to nociceptive stimuli [107]. In addition, inflammation can increase the risk of mental health problems by affecting the metabolism of neurotransmitters, and conversely, mental health issues can increase the risk of chronic pain [108].

The relationship between pain and violence could be complex; violence could be either a risk factor or a consequence, with a bidirectional relationship. It is difficult to determine which of the two causes the other because there are no longitudinal studies that track women before and after experiencing violence. At the moment it is only possible to show that the association between violence exposure and chronic pain exists and that it’s statistically significant*.*

This review represents the most systematized evidence collection between violence and chronic pain, including some diagnoses not yet considered by previous reviews, and can also evaluate the two subcategories of pelvic pain and fibromyalgia. Unfortunately, the results of the subcategories are less consistent than the general results. This may be due to the few included studies for each category. When the diagnostic categories are combined the achieved results are significant; to obtain the same effects from subcategories it could be necessary to increase the number of studies that have evaluated them. However, the fibromyalgia results show a positive and significant association, in line with the original hypothesis and previous studies [19, 29, 30].

The specific case of pelvic pain is controversial with some articles reporting an association with violence [27, 28] and others not [18, 109]. The first studies about gender violence and chronic pain initially considered only this subcategory but maybe we need to conduct further research on this aspect. Not only the small number of articles but also the study design may have influenced the results. A recent scoping review of Uvelli et al. [89] shows that for the genitourinary consequences of violence, it is possible to hypothesize a concomitance of biological and psychological aspects involving life quality and personal characteristics components. On the one hand, there are biological infections; on the other hand, maybe there is sexual dissatisfaction, having first sexual intercourse at an age in which there are not adequate cognitive abilities to process the experience, and painful intercourse, which can be both infectious and traumatic. This concomitance of aspects requires us to study the category of pelvic pain more extensively which in the past was believed to be the exclusive consequence of sexual abuse while today it also appears to be the consequence of psychological abuse.

### Limitations and potential bias

Our study has some limitations. Due to the unclear etiology and cause-effect relationship, it will be necessary to repeat a meta-analysis in the future when these data are available in the literature, in particular, when longitudinal studies are increased, and when there will be more clarity on which specific chronic pain diagnoses should be considered. In addition, the number of eligible studies was greater than the final number of included ones. Unfortunately, some important data was missing in the published version of these articles. Each first author was contacted by e-mail to gain missing data but no attempt was successful. Then, the chosen search strategy and the inclusion criteria may have been excluded from the analysis the paper from the Global South. Furthermore, the grey literature wasn’t considered. Finally, some excluded terms, such as “violence against women”, “sexual assault”, and “trauma”, could have detected other papers that could be included. It's possible that publication bias was an issue, so the researchers looked into it to ensure the accuracy of their findings.

Despite the limitations, this review represents the first to examine the association between violence against women, in all its forms, and chronic pain, in most of its representations.

## Conclusion

Gender violence is an underestimated public health problem but its consequences have serious short and long-term psychological and physical effects.

This review with meta-analysis shows strong associations between an adult history of violent victimization and chronic pain. This evidence should guide future studies on this type of association and be useful from a clinical point of view, not just a research one. Considering that many women don’t refer their violence, each healthcare professional working with chronic pain or in the emergency room should be careful and be prepared for these situations. Furthermore, the same attention should be paid by the health system in general, and by the institution to provide the correct path for these women.

## Data Availability

All data generated and analyzed during this study are included in this published article.
